# Multisensor Multi-Target Tracking Based on GM-PHD Using Out-Of-Sequence Measurements

**DOI:** 10.3390/s19194315

**Published:** 2019-10-05

**Authors:** Meiqin Liu, Tianyi Huai, Ronghao Zheng, Senlin Zhang

**Affiliations:** 1 State Key Laboratory of Industrial Control Technology, Hangzhou 310027, China; 2 College of Electrical Engineering, Zhejiang University, Hangzhou 310027, China; 21710159@zju.edu.cn (T.H.); rzheng@zju.edu.cn (R.Z.); slzhang@zju.edu.cn (S.Z.)

**Keywords:** out-of-sequence, multi-target tracking, random finite set, gaussian mixture probability hypothesis density

## Abstract

In this paper, we study the issue of out-of-sequence measurement (OOSM) in a multi-target scenario to improve tracking performance. The OOSM is very common in tracking systems, and it would result in performance degradation if we used it inappropriately. Thus, OOSM should be fully utilized as far as possible. To improve the performance of the tracking system and use OOSM sufficiently, firstly, the problem of OOSM is formulated. Then the classical B1 algorithm for OOSM problem of single target tracking is given. Next, the random finite set (RFS)-based Gaussian mixture probability hypothesis density (GM-PHD) is introduced. Consequently, we derived the equation for re-updating of posterior intensity with OOSM. Implementation of GM-PHD using OOSM is also given. Finally, several simulations are given, and results show that tracking performance of GM-PHD using OOSM is better than GM-PHD using in-sequence measurement (ISM), which can strongly demonstrate the effectiveness of our proposed algorithm.

## 1. Introduction

Multi-target tracking has been studied widely in recent years. It has attracted a lot of attention and is a major research hotspot in the field of target tracking. Multi-target tracking is not a simple extension of single-target tracking; it involves many situations that will not occur in single-target tracking. For example, in a multi-target tracking scenario, the number of targets is time-varying because new targets may appear and original targets may die. Therefore, a multi-target tracking algorithm needs to estimate the number of targets and the state of each target, at the same time. Because of the existence of clutter, it cannot guarantee that every measurement comes from a real target.

Multi-sensor measurements at each sampling time correspond to many different targets (or clutters). The problem of correlating measurements with targets is called data association, which is the key point and difficulty of a multi-target tracking algorithm and has been the focus of multi-target tracking research. Today, joint probability data association (JPDA) and multiple hypothesis tracking (MHT) are regarded as representative algorithms to solve data association problems [1,2,3,4]. There are also many improved algorithms based on JPDA and MHT. For example, [5] studied the JPDA filter with unknown detection probability and clutter rate, and [6] studied the problem of passive sonar tracking using MHT algorithm. It is noted that key developments of MHT over the past 40 years have been already reviewed in [7].

Probability hypothesis density (PHD) filter is a multi-target tracking algorithm based on random finite set (RFS) that has emerged in recent years. It was first proposed by Mahler in the literature [8]. This algorithm avoids the problem of data association. Thus, it can effectively solve the problem of a large amount of computation in a classical data association algorithm. A comparison of the RFS approach and traditional multi-target tracking methods has been given in [9]. Under the framework of RFS, the set of states of targets is regarded as a set-valued state, and the set of observations is regarded as a set-valued observation. Thus, RFS is the natural representation of the states of targets and observations of the targets. After the state and observation are modeled as RFS, a multi-target tracking problem can be formulated in Bayes filtering. Because multi-target Bayesian filtering involves multiple integrals, it will result in computational intractability. The PHD filter can alleviate the computational intractability because it operates on the single-target state space. However, there is no closed-form solution for PHD filter, so it is a common way to use the sequential Mento Carlo method to approximate it [10]. Another common implementation of PHD is Gaussian mixture probability hypothesis density (GM-PHD) [11], in which multiple Gaussian components are used to approximate PHD. Under the assumption of the linear Gaussian multi-target model, the analytical solution of GM-PHD can be obtained. This filtering algorithm is also adopted in this paper. In addition, these three multi-target tracking approaches mentioned above have been widely used in sonar [12,13], autonomous vehicles [14,15], fusion [16,17,18], visual target tracking [19,20], and sensor networks [21,22]. There are also some multi-target tracking algorithms and related work in the framework of a particle filter; for example, [23] introduced an efficient particle filter for multi-target tracking which solved the problem of the curse of dimensionality, [24] proposed two sampling techniques based on group importance sampling, and the problem of model selection in the framework of particle filter is studied in [25,26].

In a multi-target tracking system, target information measured by sensors is transmitted to the fusion center by the wireless network. However, due to network delay and other reasons, it is inevitable that some of measurements may arrive at the fusion center later. In this situation, we call it out-of-sequence measurement (OOSM). OOSM is a widely studied problem, and the main difficulty is how to use past measurements to update the current state. The research of one-step-lag OOSM in single target tracking is mature. The representative works based on Kalman filter framework are A1, B1, and C1 algorithms [27,28,29], where A1 is the optimal algorithm, and B1 and C1 are sub-optimal algorithms. In recent years, a particle filter has been widely used in the tracking system. Thus, OOSM based on a particle filter framework has also been extensively studied. Y-bar Shalom gives the optimal algorithm based on a particle filter [30]; however, this method requires excessive computational resources. To overcome this drawback, an efficient particle filter using OOSM is proposed in [31].

Although the OOSM problem and the multi-target tracking problem may appear in the tracking system simultaneously, there is little related work on the combination of them. Moreover, to the best of our knowledge, there is no related work about multi-target tracking based on RFS using OOSM that has been before. Obviously, in the actual tracking system, using OOSM in multi-target tracking is very common. In view of this, a novel multi-target tracking algorithm based on RFS using OOSM is proposed. When OOSM arrives, it is used to re-update the current state, instead of being discarded directly.

The structure of this paper is as follows. Section 2 gives the formulations of the B1 algorithm which is used to solve the problem of single-target tracking when OOSM arrives, and the PHD filter is also introduced in this section. Section 3 presents the main contribution of this paper, namely the formulation of re-update of GM-PHD filter using OOSM. And the implementation of GM-PHD filter using OOSM is also given in this section. To demonstrate the effectiveness of our proposed algorithm, several simulation results are given in Section 4. Finally, the analysis and conclusion are given in Section 5.

## 2. Problem Formulation

This section is arranged as follows. At first, the model of the system is given. Next, the problem of OOSM is formulated. Finally, the RFS based GM-PHD filter is summarized.

### 2.1. System Model

Consider a dynamic system as follows:

(1) $$x_{k+1}=F_{k}x_{k}+w_{k},$$

where $F_{k}$ is state transition matrix to time $t_{k+1}$ from time $t_{k}$, $x_{k}$ is the state vector, and $w_{k}$ is the process noise for this interval. The $F_{k}$ has different forms when the target adopts different motions, and specific $F_{k}$ related to our experiment is given in the Section 4. The state of target at time $t_{k}$ is given by $x_{k}={\left[x,v_{x},y,v_{y}\right]}^{T}$, where $\left(v_{x},v_{y}\right)$ represent the velocities in the x, y coordinate, and (x,y) is the target location. And the process noise $w_{k}$ is always assumed as zero-mean, white Gaussian noise:

(2) $$w_{k}∼N\left(0,Q_{k}\right),$$

where $Q_{k}=E\left\{w_{k}w_{k}^{T}\right\}$. The measurement equation of system is given as follows:

(3) $$z_{k}=H_{k}x_{k}+v_{k},$$

where $H_{k}$ is the measurement matrix of sensor; it also has different forms when sensors are different, and $v_{k}$ is the measurement noise which is always assumed as zero-mean, white Gaussian noise:

(4) $$v_{k}∼N\left(0,R_{k}\right),$$

where $R_{k}=E\left\{v_{k}v_{k}^{T}\right\}$.

### 2.2. OOSM B1 One-Step-Lag Algorithm

The OOSM’s problem can be formulated as follows. At time $t_{k}$, we use the measurement $Z_{k}$ to update the target state to get $x_{k}$; subsequently, an earlier measurement’s $Z_{d}$ with time stamp $t_{d}$ arrives, which satisfies the following in Equality (5), where $Z_{d}$ is called an one-step-lag OOSM. The goal is to re-estimate the current state $x_{k}$ with OOSM $Z_{d}$.

(5) $$t_{k-1}<t_{d}<st_{k}.$$

**Figure 1:**
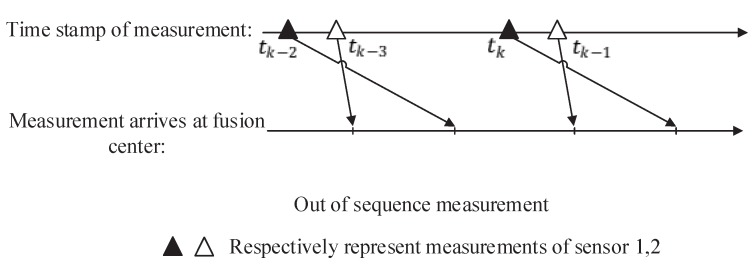
Out-of-sequence measurement (OOSM).

Applying Equation (1), we can get the backward transition equation as follows:

(6) $$x_{k}=F_{k,d}x_{d}+w_{k,d},$$

where $x_{d}$ is the state of target at time *d*, F(k,d) is the state transition matrix to time $t_{k}$ from time $t_{d}$, and $w_{k,d}$ is the process noise during this interval. And then, we can get the backward transition equation from (5):

(7) $$x_{d}=F_{d,k}\left(x_{k}-w_{k,d}\right),$$

where $F_{d,k}$ is the backward transition matrix, and it is also the inverse matrix of $F_{k,d}$, which means $F_{d,k}=F_{k,d}^{-1}$. For a one-lag measurement problem, in which $t_{k-1}<t_{d}<t_{k}$, the schematic diagram is shown in Figure 1.

Based on the LMMSE criterion, using the basic equation of linear estimation, we can get the backward prediction of the state from $t_{k}$ to $t_{d}$ as follows:

(8) $${\hat{x}}_{d|k}=F_{k,d}\left({\hat{x}}_{k|k}-{\hat{w}}_{k,d|k}\right),$$

where ${\hat{w}}_{k,d|k}$ is the estimation of process noise of backward prediction, and it is assumed to be zero in the sub-optimal B1 algorithm, which means under this situation ${\hat{w}}_{k,d|k}=0$, and based on this point, we can get the following equation:

(9) $${\hat{x}}_{d|k}=F_{k,d}{\hat{x}}_{k|k}.$$

Then the covariance matrix of backward state prediction can be calculated as follows:

(10) $$P_{d|k}=F_{k,d}\left[P_{k|k}+P_{k,d|k}^{ww}-P_{k,d|k}^{xw}-{\left(P_{k,d|k}^{xw}\right)}^{T}\right]F_{k,d}^{T}.$$

The related covariance matrix in the Equation (10) can be calculated as follows:

(11) $$P_{k,d|k}^{ww}=Q_{k,d},$$

(12) $$P_{k,d|k}^{xw}=Q_{k,d}-P_{k|k-1}H_{k}^{T}S_{k}^{-1}H_{k}Q_{k,d},$$

(13) $$P_{k|k}=P_{k|k-1}-P_{k|k-1}H_{k}^{T}S_{k}^{-1}H_{k}P_{k|k-1}.$$

Here, we just give the equations of the B1 algorithm, and the derivation of the equations in detail can be found in [27]. The covariance matrix of the measurement of backward state prediction is

(14) $$S_{d}=H_{d}P_{d|k}H_{d}^{T}+R_{d}.$$

The covariance matrix between state $x_{k}$ and measurement $z_{d}$ is

(15) $$P_{k,d|k}^{xz}=\left(P_{k|k}-P_{k,d|k}^{xw}\right)F_{d,k}^{T}H_{d}^{T}.$$

Then we can calculate the filter gain as follows:

(16) $$K_{d}=P_{k,d|k}^{xz}{\left(S_{d}\right)}^{-1};$$

therefore, the re-update of current state ${\hat{x}}_{k|k}$ using the OOSM $z_{d}$ is

(17) $${\hat{x}}_{k|d}={\hat{x}}_{k|k}+K_{d}\left(z_{d}-H_{d}{\hat{x}}_{k|d}\right).$$

### 2.3. GM-PHD Filter

As mentioned in the previous section, the multi-target tracking algorithm based on random sets effectively avoids the problem of data association. Moreover, it does not need to know the number of targets in advance. In a word, it brings a new idea to solve the problem of multi-target tracking.

In multi-target tracking scenarios, the birth and death of the target should also be considered except from the target motion. Thus, it is necessary not only to estimate the state of multiple targets, but also to estimate the number of targets. This means that the number of elements in the multi-target state set and measurement set is variable. So, in a multi-target situation, the states of targets and measurements can be represented by the following:

(18) $$\begin{array}{c} X_{k}=\left\{x_{k}^{1},\ldots ,x_{k}^{n(k)}\right\}\in F\left(E_{s}\right), \end{array}$$

(19) $$\begin{array}{c} Z_{k}=\left\{z_{k}^{1},\ldots ,z_{k}^{m(k)}\right\}\in F\left(E_{o}\right), \end{array}$$

where n(k) and m(k) are the number of targets and the number of measurements at time $t_{k}$, respectively, and $F(E_{s})$ and $F(E_{o})$ are the multi-target state space and measurement space.

In the multi-target scenario, the birth and death of the target should also be considered separately from the target motion. At time $t_{k-1}$, each target either survives at time $t_{k}$ with the probability $p_{s,k}$, or dies with probability $1-p_{s,k}$. At time $t_{k}$, a new target may arise either by newborn or by spawning from a target at time $t_{k-1}$. Therefore, the multi-target state of next time $t_{k}$ can be modeled by the following RFS:

(20) $$\begin{array}{c} X_{k}=S_{k}\left(X_{k-1}\right)\cup B_{k}\left(X_{k-1}\right)\cup \Gamma_{k}\left(X_{k-1}\right), \end{array}$$

where $S_{k}\left(X_{k-1}\right)$ is the RFS of multi-target state at time $t_{k}$, which evolves from time $t_{k-1}$, $B_{k}\left(X_{k-1}\right)$ is the RFS of the newborn target, and $\Gamma_{k}\left(X_{k-1}\right)$ is RFS of targets spawned from the previous state. Due to the existence of clutters in measurement, the RFS of measurement can be modeled as:

(21) $$\begin{array}{c} Z_{k}=\Theta_{k}\left(X_{k-1}\right)\cup K_{k}\left(X_{k-1}\right), \end{array}$$

where $\Theta_{k}\left(X_{k-1}\right)$ is the RFS of measurement of targets, and $K_{k}\left(X_{k-1}\right)$ is the RFS of measurement of clutters.

The PHD filter is a typical RFS-based multi-target tracking algorithm. However, there is no closed form solution to PHD recursion, in general. But the PHD recursion has a closed-form solution in a certain class of situation, in which the multi-target model satisfies the linear Gaussian assumption. In this situation, the Gaussian mixture is used to approximate the intensity.

For clarity, the Gaussian mixture PHD (GM-PHD) filter is summarized in this paper, and readers can find more details in [11]. Under linear Gaussian assumption, the intensity at time $t_{k-1}$ can be represented by the following equation:

(22) $$\begin{array}{c} v_{k-1}\left(x\right)=\sum_{i=1}^{J_{k-1}}\omega_{k-1}^{\left(i\right)}N\left(x;m_{k-1}^{\left(i\right)},P_{k-1}^{\left(i\right)}\right). \end{array}$$

Then, the predicted intensity is given as follows:

(23) $$\begin{array}{cc} v_{k|k-1}\left(x\right)= & v_{S,k|k-1}\left(x\right)+v_{\beta ,k|k-1}\left(x\right)+v_{\gamma ,k|k-1}\left(x\right)\\ = & \sum_{i=1}^{J_{k|k-1}}\omega_{k|k-1}^{\left(i\right)}N\left(x;m_{k|k-1}^{\left(i\right)},P_{k|k-1}^{\left(i\right)}\right), \end{array}$$

where $v_{S,k|k-1}\left(x\right)$, $v_{\beta ,k|k-1}\left(x\right)$, $v_{\gamma ,k|k-1}\left(x\right)$ represent intensity of survival target, intensity of new target spawned by the previous target, and intensity of newborn target, respectively. These three intensities are also Gaussian mixture, and their specific expressions can be found in [11]. Next, we can get the posterior intensity, which is given as follows:

(24) $$\begin{array}{c} v_{k}\left(x\right)=\left(1-p_{D,k}\right)v_{k|k-1}\left(x\right)+\sum_{z\in Z_{k}}v_{D,k}\left(x;z\right), \end{array}$$

(25) $$\begin{array}{c} v_{D,k}\left(x;z\right)=\sum_{i=1}^{J_{k|k-1}}\omega_{k}^{\left(i\right)}\left(z\right)N\left(x;m_{k|k}^{\left(i\right)},P_{k|k}^{\left(i\right)}\right), \end{array}$$

where $p_{D,k}$ is the probability of detect, $v_{D,k}\left(x;z\right)$ is the update item of predicted intensity, and the expression of $\omega_{k}^{\left(i\right)}\left(z\right)$, $m_{k|k}^{\left(i\right)}$, $P_{k|k}^{\left(i\right)}$ can be found in [11]. Finally, the pruning and merging is performed, and readers can also find the details in [11].

## 3. Multi-Target OOSM Tracking Algorithm

**Figure 2:**
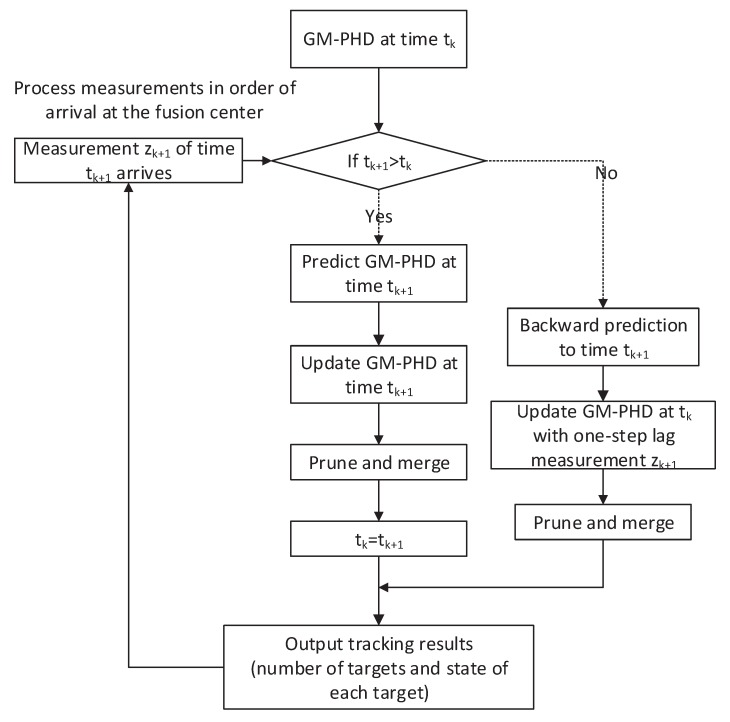
Flowchart of proposed algorithm. GM-PHD = Gaussian mixture probability hypothesis density.

In the above two sections, the problems of OOSM and multi-target tracking based on RFS were discussed. In fact, these two issues have been widely studied. And the issue of multi-target tracking using OOSM has also attracted a lot of attention recently. However, to the best of our knowledge, there is no relative work about RFS-based multi-target tracking using OOSM. In the following, a novel multi-target tracking algorithm based on RFS is proposed, in which OOSM is used to re-update the posterior intensity. As we can see from Equations (9) and (12), predicted covariance $P_{k|k-1}$ is needed to calculate the retrodiction covariance $P_{d|k}$. However, in the GM-PHD algorithm, after pruning and merging, there is only posterior information left in the Gaussian component. In this situation, OOSM cannot be used to re-update the posterior intensity. Therefore, when measurement arrives, the judgment of whether the measurement is a delay should be executed first. If the timestamp of measurement shows that the measurement is in sequence, the update process of the GM-PHD filter is performed. On the contrary, if the measurement is a delay, our proposed algorithm is performed to re-update the multi-target state at time $t_{k}$. The flowchart of the proposed algorithm is given in Figure 2. Recalling the update equation of GM-PHD filter, substituting Equations (23) and (25) into update Equation (24), then the update equation can be rewritten as follows:

(26) $$\begin{array}{cc} v_{k}\left(x\right) & =\left(1-p_{D,k}\right)v_{k|k-1}\left(x\right)+\sum_{z\in Z_{k}}\sum_{i=1}^{J_{k|k-1}}\omega_{k}^{\left(i\right)}\left(z\right)N\left(x;m_{k|k}^{\left(i\right)}\left(z\right),P_{k|k}^{\left(i\right)}\right)\\ \; & =\left(1-p_{D,k}\right)\sum_{i=1}^{J_{k|k-1}}\omega_{k|k-1}^{\left(i\right)}N\left(x;m_{k|k-1}^{\left(i\right)},P_{k|k-1}^{\left(i\right)}\right)+\sum_{i=1}^{|Z_{k}|J_{k|k-1}}\omega_{k}^{\left(i\right)}N\left(x;m_{k|k}^{\left(i\right)},P_{k|k}^{\left(i\right)}\right), \end{array}$$

where the $|Z_{k}|$ is the number of elements of set $Z_{k}$. In Equation (26), the first item in the right side is the prior intensity, which is associated with the estimated state ${\hat{x}}_{k|k-1}$. It also represents targets which are not detected, and we refer to it herein a ’prior target’. The second item represents detected targets, which we refer to herein as ’posterior target’.

**Figure 3:**
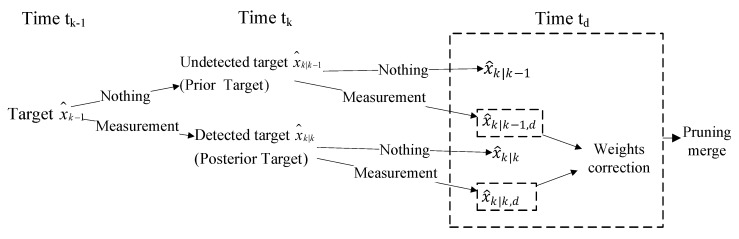
OOSM.

The ’prior target’ is the target which is not detected at time $t_{k}$, but when OOSM arrives, it may contain information about the ’prior target’. Therefore, when OOSM arrives, the estimated state ${\hat{x}}_{k|k-1}$ associated with ’prior target’ should be updated. Analogously, the ’posterior target’ is also needed to re-update using OOSM, and the weights of Gaussian components also need to be modified. The diagram of the classification processing algorithm using OOSM is shown in Figure 3.

In the following, the derivation of the proposed algorithm is given.

### 3.1. Backward State Prediction

For simplicity, we assumed that there is no targets newborning and spawning from exist targets during time $t_{d}$ to $t_{k}$, which means the number of targets at time $t_{k}$ and time $t_{d}$ are the same. The multi-target state at time $t_{d}$ can be represented by the following:

(27) $$\begin{array}{c} X_{d}=\underset{x\in X_{k|k-1}\cup X_{D,k}}{⋃}S_{d|k}\left(x\right), \end{array}$$

where $S_{d|k}\left(x\right)$ is the RFS of multi-target state at time $t_{d}$ which evolve from time $t_{k}$, $X_{k|k-1}$ is the RFS of undetected targets’ state, and $X_{D,k}$ is the RFS of detected targets’ state. The intensity at time $t_{d}$ can be divided into two items. One is retrodicted from the undetected intensity $\left(1-p_{D,k}\right)v_{k|k-1}\left(x\right)$. The other is retrodicted from the detected intensity $\sum_{z\in Z_{k}}v_{D,k}\left(x;z\right)$. Applying retrodiction Equations (9) and (10) of the B1 algorithm, the intensity retrodicted from the undetected intensity is given as follows:

(28) $$\begin{array}{cc} v_{d|k-1}\left(x\right)=v_{S,d|k-1}= & p_{S,d}\left(1-p_{D,k}\right)\sum_{i=1}^{J_{k|k-1}}\omega_{k|k-1}^{\left(i\right)}N\left(x;m_{d|k-1}^{\left(i\right)},P_{d|k-1}^{\left(i\right)}\right)\\ = & \sum_{i=1}^{J_{k|k-1}}\omega_{d|k-1}^{\left(i\right)}N\left(x;m_{d|k-1}^{\left(i\right)},P_{d|k-1}^{\left(i\right)}\right), \end{array}$$

where

(29) $$\begin{array}{c} \omega_{d|k-1}^{\left(i\right)}=\left(1-p_{D,k}\right)p_{S,d}\omega_{k|k-1}^{\left(i\right)},\;m_{d|k-1}^{\left(i\right)}=F_{d,k}m_{k|k-1}^{\left(i\right)}, \end{array}$$

(30) $$P_{d|k-1}^{\left(i\right)}=F_{d,k}\left[P_{k|k-1}^{\left(i\right)}+P_{k,d|k-1}^{ww(i)}-P_{k,d|k-1}^{xw(i)}-{\left(P_{k,d|k-1}^{xw(i)}\right)}^{T}\right]F_{d,k}^{T}$$

where $P_{k,d|k-1}^{ww(i)}$ is the *i*th Gaussian component’s covariance of process noise from time $t_{d}$ to time $t_{k}$, and $P_{k,d|k}^{xw(i)}$ is the *i*th Gaussian component’s cross-covariance between state and process noise. They can be represented as follows:

(31) $$\begin{array}{cc} P_{k,d|k-1}^{ww(i)} & =\mathrm{cov}\left\{w_{k,d}|Z^{k-1}\right\}=Q_{k,d}, \end{array}$$

(32) $$\begin{array}{cc} P_{k,d|k-1}^{xw(i)} & =\mathrm{cov}\{w_{k,d},x_{k}|Z^{k-1}\}\\ \; & =E\{w_{k,d}{\left[x_{k}-{\hat{x}}_{k|k-1}\right]}^{T}|Z^{k-1}\}\\ \; & =E\{w_{k,d}{\left[F_{k,d}x_{d}+w_{k,d}-{\hat{x}}_{k|k-1}\right]}^{T}|Z^{k-1}\}\\ \; & =E\{w_{k,d}w_{k,d}^{T}|Z^{k-1}\}\\ \; & =Q_{k,d}. \end{array}$$

Substituting Equations (31) and (32) into (30), we can rewrite Equation (30) as follows:

(33) $$\begin{array}{cc} P_{d|k-1}^{\left(i\right)} & =F_{d,k}\left[P_{k|k-1}^{\left(i\right)}+P_{k,d|k-1}^{ww(i)}-P_{k,d|k}^{xw(i)}-{\left(P_{k,d|k}^{xw(i)}\right)}^{T}\right]F_{d,k}^{T}\\ \; & =F_{d,k}\left[P_{k|k-1}^{\left(i\right)}-Q_{k,d}\right]F_{d,k}^{T}. \end{array}$$

Analogously, we can easily get the intensity retrodicted from the detected intensity:

(34) $$\begin{array}{cc} v_{d|k}\left(x\right)=v_{S,d|k}= & p_{S,d}\sum_{i=1}^{|Z_{k}|J_{k|k-1}}\omega_{k|k}^{\left(i\right)}N\left(x;m_{d|k}^{\left(i\right)},P_{d|k}^{\left(i\right)}\right)\\ = & \sum_{i=1}^{|Z_{k}|J_{k|k-1}}\omega_{d|k}^{\left(i\right)}N\left(x;m_{d|k}^{\left(i\right)},P_{d|k}^{\left(i\right)}\right), \end{array}$$

where

(35) $$\begin{array}{c} \omega_{d|k}^{\left(i\right)}=p_{S,d}\omega_{k|k}^{\left(i\right)},\;m_{d|k}^{\left(i\right)}=F_{d,k}m_{k|k}^{\left(i\right)}, \end{array}$$

(36) $$P_{d|k}^{\left(i\right)}=F_{d,k}\left[P_{k|k}^{\left(i\right)}+P_{k,d|k}^{ww,(i)}-P_{k,d|k}^{xw,(i)}-{\left(P_{k,d|k}^{xw,(i)}\right)}^{T}\right]F_{d,k}^{T},$$

where $P_{k|k}^{\left(i\right)}$, $P_{k,d|k}^{ww,(i)}$, and $P_{k,d|k}^{xw,(i)}$ can be derived from Equations (10)–(12). And the pseudo-code for backward state prediction is given in Algorithm 1.

**Algorithm 1** Backward state prediction
Input ${\left\{w_{k}^{\left(i\right)},m_{k}^{\left(i\right)},P_{k}^{\left(i\right)}\right\}}_{i=1}^{J_{k|k}}$ = ${\left\{w_{k|k-1}^{\left(i\right)},m_{k|k-1}^{\left(i\right)},P_{k|k-1}^{\left(i\right)}\right\}}_{i=1}^{J_{k|k-1}}\cup {\left\{w_{k|k}^{\left(i\right)},m_{k|k}^{\left(i\right)},P_{k|k}^{\left(i\right)}\right\}}_{i=1}^{|Z_{k}|J_{k|k-1}}$, and the out of sequence measurement $Z_{d}$Step 1. Backward prediction
**for** $i=1,2,\ldots ,J_{k|k-1}$ **do**
 $\omega_{d|k-1}^{\left(j\right)}=\left(1-p_{D,d}\right)p_{S,d}\omega_{k|k-1}^{\left(i\right)},m_{d|k-1}^{\left(i\right)}=F_{d,k}m_{k|k-1}^{\left(i\right)}$
 $P_{d|k-1}^{\left(i\right)}=F_{d,k}\left[P_{k|k-1}^{\left(i\right)}+P_{k,d|k-1}^{ww,(i)}-P_{k,d|k-1}^{xw,(i)}-{\left(P_{k,d|k-1}^{xw,(i)}\right)}^{T}\right]F_{d,k}^{T}$
**end for**

**for** $i=1,\ldots ,|Z_{k}|J_{k|k-1}$ **do**
 $\omega_{d|k}^{\left(j\right)}=p_{S,d}\omega_{k|k}^{\left(i\right)},m_{d|k}^{\left(i\right)}=F_{d,k}m_{k|k}^{\left(i\right)}$ $P_{d|k}^{\left(i\right)}=F_{d,k}\left[P_{k|k}^{\left(i\right)}+P_{k,d|k}^{ww,(i)}-P_{k,d|k}^{xw,(i)}-{\left(P_{k,d|k}^{xw,(i)}\right)}^{T}\right]F_{d,k}^{T}$
**end for**
Output ${\left\{w_{d|k-1}^{\left(i\right)},m_{d|k-1}^{\left(i\right)},P_{d|k-1}^{\left(i\right)}\right\}}_{i=1}^{J_{k|k-1}}\cup {\left\{w_{d|k}^{\left(i\right)},m_{d|k}^{\left(i\right)},P_{d|k}^{\left(i\right)}\right\}}_{i=1}^{|Z_{k}|J_{k|k-1}}$


### 3.2. Re-Update with OOSM

It has been mentioned in Equation (26) that intensity $v_{k}\left(x\right)$ can be divided into two items. Therefore, when OOSM arrives, it is used for re-updating these two items, respectively, by using OOSM, and we can get the following re-update equation:

(37) $$\begin{array}{cc} v_{k|d}\left(x\right)= & \left(1-p_{D,d}\right)\left\{\left[1-p_{D,k}\right]v_{k|k-1}\left(x\right)+\sum_{z\in Z_{k}}v_{D,k}\left(x;z\right)\right\}+\sum_{z\in Z_{d}}\left\{v_{D,k|k-1,d}\left(x;z\right)+v_{D,k|k,d}\left(x;z\right)\right\}\\ = & \left(1-p_{D,d}\right)v_{k|k}+\sum_{z\in Z_{d}}\left\{v_{D,k|k-1,d}\left(x;z\right)+v_{D,k|k,d}\left(x;z\right)\right\}\; \end{array}$$

where the first item represents the undetected intensity at time $t_{d}$, and the second item represents the detected intensity at time $t_{d}$. And intensities $v_{D,k|k-1,d}\left(x;z\right)$, $v_{D,k|k,d}\left(x;z\right)$ are given by:

(38) $$\begin{array}{c} v_{D,k|k-1,d}\left(x;z\right)=\sum_{i=1}^{J_{k|k-1}}\omega_{k|k-1,d}^{\left(i\right)}N\left(x;m_{k|k-1,d}^{\left(i\right)},P_{k|k-1,d}^{\left(i\right)}\right), \end{array}$$

(39) $$\begin{array}{c} m_{k|k-1,d}^{\left(i\right)}=m_{k|k-1}^{\left(i\right)}+K_{d,k-1}^{\left(i\right)}\left(z-H_{d}m_{d|k-1}^{\left(i\right)}\right), \end{array}$$

(40) $$\begin{array}{c} P_{k|k-1,d}^{\left(i\right)}=P_{k|k-1}^{\left(i\right)}-P_{k,d|k-1}^{xz,(i)}{\left(S_{d|k-1}^{\left(i\right)}\right)}^{-1}{\left(P_{k,d|k-1}^{xz,(i)}\right)}^{T}, \end{array}$$

(41) $$\begin{array}{c} v_{D,k|k,d}\left(x;z\right)=\sum_{i=1}^{|Z_{k}|J_{k|k-1}}\omega_{k|k,d}^{\left(i\right)}N\left(x;m_{k|d}^{\left(i\right)},P_{k|d}^{\left(i\right)}\right), \end{array}$$

(42) $$\begin{array}{c} m_{k|d}^{\left(i\right)}=m_{k|k}^{\left(i\right)}+K_{d,k}^{\left(i\right)}\left(z-H_{d}m_{d|k}^{\left(i\right)}\right), \end{array}$$

(43) $$\begin{array}{c} P_{k|k,d}^{\left(i\right)}=P_{k|k}^{\left(i\right)}-P_{k,d|k}^{xz,(i)}{\left(S_{d|k}^{\left(i\right)}\right)}^{-1}{\left(P_{k,d|k}^{xz,(i)}\right)}^{T}, \end{array}$$

where $K_{d,k-1}^{\left(i\right)}$, $S_{d|k-1}^{\left(i\right)}$, $K_{d,k}^{\left(i\right)}$ and $S_{d|k}^{\left(i\right)}$ can be derived from the Equations (13) and (15). The pseudo-code is given in Algorithm 2. And weights $\omega_{k|k-1,d}^{\left(i\right)}$, $\omega_{k|k,d}^{\left(i\right)}$ are given in the next section.

**Algorithm 2** Re-update using OOSM
Input the output of backward state prediction ${\left\{w_{d|k-1}^{\left(i\right)},m_{d|k-1}^{\left(i\right)},P_{d|k-1}^{\left(i\right)}\right\}}_{i=1}^{J_{k|k-1}}\cup {\left\{w_{d|k}^{\left(i\right)},m_{d|k}^{\left(i\right)},P_{d|k}^{\left(i\right)}\right\}}_{i=1}^{|Z_{k}|J_{k|k-1}}$Step 1. PHD update components
**for** $i=1,2,\ldots ,J_{k|k-1}$ **do**
 $P_{k,d|k-1}^{xz,(i)}=\left(P_{k|k-1}-Q_{k,d}\right)F_{d,k}^{T}H_{d}^{T}$
 $S_{d|k-1}^{\left(i\right)}=H_{d}P_{d|k-1}^{\left(i\right)}H_{d}^{T}+R_{d}$
 $K_{k,d|k-1}^{\left(i\right)}=P_{k,d|k-1}^{xz,(i)}{\left(S_{d|k-1}^{\left(i\right)}\right)}^{-1}$

**end for**

**for** $i=J_{k|k-1}+1,\ldots ,\left|Z_{k}\right|J_{k|k-1}$ **do**
 $P_{k,d|k}^{xw,(i)}=Q_{k,d}-P_{k|k-1}^{\left(i\right)}H_{k}^{T}{\left(S_{k}^{\left(i\right)}\right)}^{-1}H_{k}Q_{k,d}$
 $P_{k,d|k}^{xz,(i)}=\left(P_{k|k}^{\left(i\right)}-P_{k,d|k}^{xw,(i)}\right)F_{d,k}^{T}H_{d}^{T}$
 $S_{d|k}^{\left(i\right)}=H_{d}P_{d|k}^{\left(i\right)}H_{d}^{T}+R_{d}$
 $K_{k,d|k}^{\left(i\right)}=P_{k,d|k}^{xz,(i)}{\left(S_{d|k}^{\left(i\right)}\right)}^{-1}$

**end for**
Step 2. Re-update using OOSM
**for** $l=0,1,\ldots ,|Z_{d}|-1$ **do**
 **for**$j=1,2,\ldots ,J_{k|k-1}$ **do**  $\omega_{k|k-1,d}^{\left(lJ_{k|k-1}+j\right)}=p_{D,d}\omega_{d|k-1}^{\left(j\right)}N\left(z^{\left(l+1\right)};H_{d}m_{d|k-1}^{\left(j\right)},S_{d|k-1}^{\left(j\right)}\right)$
  $m_{k|k-1,d}^{\left(lJ_{k|k-1}+j\right)}=m_{k|k-1}^{\left(j\right)}+K_{k,d|k-1}^{\left(j\right)}\left(z-H_{d}m_{d|k-1}^{\left(j\right)}\right)$  $P_{k|k-1,d}^{\left(lJ_{k|k-1}+j\right)}=P_{k|k-1}^{\left(j\right)}-P_{k,d|k-1}^{xz,(j)}{\left(S_{d|k-1}^{\left(j\right)}\right)}^{-1}{\left(P_{k,d|k-1}^{xz,(j)}\right)}^{T}$ **end for** **for** $j=1,2,\ldots ,|Z_{k}|J_{k|k-1}$ **do**  $\omega_{k|k,d}^{\left(l|Z_{k}|J_{k|k-1}+j\right)}=p_{D,d}\omega_{d|k}^{\left(j\right)}N\left(z^{\left(l+1\right)};H_{d}m_{d|k}^{\left(j\right)},S_{d|k}^{\left(j\right)}\right)$  $m_{k|d}^{\left(l|Z_{k}|J_{k|k-1}+j\right)}=m_{k|k}^{\left(j\right)}+K_{k,d|k}^{\left(i\right)}\left(z-H_{d}m_{d|k}^{\left(i\right)}\right)$
  $P_{k|d}^{\left(l|Z_{k}|J_{k|k-1}+j\right)}=P_{k|k}^{\left(j\right)}-P_{k,d|k}^{xz,(j)}{\left(S_{d|k}^{\left(j\right)}\right)}^{-1}{\left(P_{k,d|k}^{xz,(j)}\right)}^{T}$
 **end for**
**end for**
Output ${\left\{\omega_{k|k-1,d}^{\left(i\right)},m_{k|k-1,d}^{\left(i\right)},P_{k|k-1,d}^{\left(i\right)}\right\}}_{i=1}^{|Z_{d}|J_{k|k-1}}\cup {\left\{w_{k|k,d}^{\left(i\right)},m_{k|k,d}^{\left(i\right)},P_{k|k,d}^{\left(i\right)}\right\}}_{i=1}^{|Z_{d}\left|\right|Z_{k}|J_{k|k-1}}$


### 3.3. Weights Correction

In the above section, we get the intensities that are re-updated with OOSM. These two intensities are re-updated using the same measurement $Z_{d}$, which is different from Equation (39). Weights of the intensities need to be corrected, and corrected weights are given as follows:

Prior target:

(44) $$\begin{array}{c} \omega_{k|k-1,d}^{\left(i\right)}=\frac{p_{D,k}\omega_{d|k-1}^{\left(i\right)}q_{d}^{\left(i\right)}\left(z\right)}{\kappa_{k}\left(z\right)+p_{D,k}\left(\sum_{j=1}^{J_{k|k-1}}\omega_{d|k-1}^{\left(j\right)}q_{d|k-1}^{\left(j\right)}\left(z\right)+\sum_{l=J_{k|k-1}+1}^{|Z_{k}|J_{k|k-1}}\omega_{k|d}^{\left(l\right)}q_{d|k}^{\left(l\right)}\left(z\right)\right)}. \end{array}$$

Posterior target:

(45) $$\begin{array}{c} \omega_{k|k,d}^{\left(i\right)}=\frac{p_{D,k}\omega_{k|d}^{\left(i\right)}q_{d}^{\left(i\right)}\left(z\right)}{\kappa_{k}\left(z\right)+p_{D,k}\left(\sum_{j=1}^{J_{k|k-1}}\omega_{d|k-1}^{\left(j\right)}q_{d|k-1}^{\left(j\right)}\left(z\right)+\sum_{l=J_{k|k-1}+1}^{|Z_{k}|J_{k|k-1}}\omega_{d|k}^{\left(l\right)}q_{d|k}^{\left(l\right)}\left(z\right)\right)}, \end{array}$$

where

(46) $$\begin{array}{c} q_{d|k-1}^{\left(j\right)}\left(z\right)=N\left(z;H_{d}m_{d|k-1}^{\left(j\right)},H_{d}P_{d|k-1}^{\left(j\right)}H_{d}^{T}+R_{d}\right), \end{array}$$

(47) $$\begin{array}{c} q_{d|k}^{\left(l\right)}\left(z\right)=N\left(z;H_{d}m_{d|k}^{\left(l\right)},H_{d}P_{d|k}^{\left(l\right)}H_{d}^{T}+R_{d}\right). \end{array}$$

For completeness, the pseudo-code for GM-PHD using OOSM is given in Algorithm 3.

**Algorithm 3** Weight correction
Input ${\left\{w_{k|k-1}^{\left(i\right)},m_{k|k-1}^{\left(i\right)},P_{k|k-1}^{\left(i\right)}\right\}}_{i=1}^{J_{k|k-1}}\cup {\left\{w_{k|k}^{\left(i\right)},m_{k|k}^{\left(i\right)},P_{k|k}^{\left(i\right)}\right\}}_{i=1}^{|Z_{k}|J_{k|k-1}}\cup {\left\{\omega_{k|k-1,d}^{\left(j\right)},m_{k|k-1,d}^{\left(i\right)},P_{k|k-1,d}^{\left(i\right)}\right\}}_{i=1}^{|Z_{d}|J_{k|k-1}}\cup {\left\{w_{k|k,d}^{\left(i\right)},m_{k|k,d}^{\left(i\right)},P_{k|k,d}^{\left(i\right)}\right\}}_{i=1}^{|Z_{d}\left|\right|Z_{k}|J_{k|k-1}}$Step 1. Weight Correction
**for** $l=0,1,\ldots ,|Z_{d}|-1$ **do**
 **for** $i=1,2,\ldots ,J_{k|k-1}$ **do**  $\omega_{k|k-1,d}^{\left(lJ_{k|k-1}+i\right)}=\frac{\omega_{k|k-1,d}^{\left(i\right)}}{\kappa_{k}\left(z\right)+\sum_{i=1}^{|Z_{d}|J_{k|k-1}}\omega_{k|k-1,d}^{\left(i\right)}+\sum_{i=1}^{|Z_{k}\left|\right|Z_{d}|J_{k|k-1}}\omega_{k|k,d}^{\left(i\right)}}$ **end for** **for** $i=1,2,\ldots ,|Z_{k}|J_{k|k-1}$ **do**  $\omega_{k|k,d}^{\left(l|Z_{k}|J_{k|k-1}+i\right)}=\frac{\omega_{k|k,d}^{\left(i\right)}}{\kappa_{k}\left(z\right)+\sum_{i=1}^{|Z_{d}|J_{k|k-1}}\omega_{k|k-1,d}^{\left(i\right)}+\sum_{i=1}^{|Z_{k}\left|\right|Z_{d}|J_{k|k-1}}\omega_{k|k,d}^{\left(i\right)}}$
 **end for**
**end for**
Output ${\left(1-p_{D,d}\right)}^{2}w_{k|k-1}^{\left(i\right)},m_{k|k-1}^{\left(i\right)},P_{k|k-1}^{\left(i\right)}{\}}_{i=1}^{J_{k|k-1}}\cup {\left\{\left(1-p_{D,d}\right)w_{k|k}^{\left(i\right)},m_{k|k}^{\left(i\right)},P_{k|k}^{\left(i\right)}\right\}}_{i=1}^{|Z_{k}|J_{k|k-1}}\cup {\left\{\omega_{k|k-1,d}^{\left(j\right)},m_{k|k-1,d}^{\left(i\right)},P_{k|k-1,d}^{\left(i\right)}\right\}}_{i=1}^{|Z_{d}|J_{k|k-1}}\cup {\left\{w_{k|k,d}^{\left(i\right)},m_{k|k,d}^{\left(i\right)},P_{k|k,d}^{\left(i\right)}\right\}}_{i=1}^{|Z_{d}\left|\right|Z_{k}|J_{k|k-1}}$


**Algorithm 4** RFS-based multi-target tracking using OOSM
Input${\left\{w_{k}^{\left(i\right)},m_{k}^{\left(i\right)},P_{k}^{\left(i\right)}\right\}}_{i=1}^{J_{k|k}}$ = ${\left\{w_{k|k-1}^{\left(i\right)},m_{k|k-1}^{\left(i\right)},P_{k|k-1}^{\left(i\right)}\right\}}_{i=1}^{J_{k|k-1}}\cup {\left\{w_{k|k}^{\left(i\right)},m_{k|k}^{\left(i\right)},P_{k|k}^{\left(i\right)}\right\}}_{i=1}^{|Z_{k}|J_{k|k-1}}$, and the out of sequence measurement $Z_{d}$
i=0
Step 1. Backward prediction
**for** $j=1,2,\ldots ,J_{k|k-1}$ **do**
 $\omega_{d|k-1}^{\left(j\right)}=p_{S,d}\omega_{k|k-1}^{\left(j\right)},m_{d|k-1}^{\left(j\right)}=F_{d,k}m_{k|k-1}^{\left(j\right)}$ $P_{d|k-1}^{\left(i\right)}=F_{d,k}\left[P_{k|k-1}^{\left(i\right)}+P_{k,d|k-1}^{ww,(i)}-P_{k,d|k-1}^{xw,(i)}-{\left(P_{k,d|k-1}^{xw,(i)}\right)}^{T}\right]F_{d,k}^{T}$
**end for**

**for** $j=1,\ldots ,|Z_{k}|J_{k|k-1}$ **do**
 $\omega_{d|k}^{\left(j\right)}=p_{S,d}\omega_{k|k}^{\left(j\right)},m_{d|k}^{\left(j\right)}=F_{d,k}m_{k|k}^{\left(j\right)}$ $P_{d|k}^{\left(i\right)}=F_{d,k}\left[P_{k|k}^{\left(i\right)}+P_{k,d|k}^{ww,(i)}-P_{k,d|k}^{xw,(i)}-{\left(P_{k,d|k}^{xw,(i)}\right)}^{T}\right]F_{d,k}^{T}$

**end for**
Step 2. PHD update components
**for** $j=1,2,\ldots ,J_{k|k-1}$ **do**
 $P_{k,d|k-1}^{xz,(i)}=\left(P_{k|k-1}-Q_{k,d}\right)F_{d,k}^{T}H_{d}^{T}$ $S_{d|k-1}^{\left(i\right)}=H_{d}P_{d|k-1}^{\left(i\right)}H_{d}^{T}+R_{d}$ $K_{k,d|k-1}^{\left(i\right)}=P_{k,d|k-1}^{xz,(i)}{\left(S_{d|k-1}^{\left(i\right)}\right)}^{-1}$

**end for**

**for** $j=J_{k|k-1}+1,\ldots ,\left|Z_{k}\right|J_{k|k-1}$ **do**
 $P_{k,d|k}^{xw,(i)}=Q_{k,d}-P_{k|k-1}^{\left(i\right)}H_{k}^{T}{\left(S_{k}^{\left(i\right)}\right)}^{-1}H_{k}Q_{k,d}$ $P_{k,d|k}^{xz,(i)}=\left(P_{k|k}^{\left(i\right)}-P_{k,d|k}^{xw,(i)}\right)F_{d,k}^{T}H_{d}^{T}$ $S_{d|k}^{\left(i\right)}=H_{d}P_{d|k}^{\left(i\right)}H_{d}^{T}+R_{d}$
 $K_{k,d|k}^{\left(i\right)}=P_{k,d|k}^{xz,(i)}{\left(S_{d|k}^{\left(i\right)}\right)}^{-1}$

**end for**
Step 3. Re-update using OOSM
**for** $l=0,1,\ldots ,|Z_{d}|-1$ **do**
 **for** $j=1,2,\ldots ,J_{k|k-1}$ **do**
  $\omega_{k|k-1,d}^{\left(lJ_{k|k-1}+j\right)}=p_{D,d}\omega_{d|k-1}^{\left(j\right)}N\left(z^{\left(l+1\right)};H_{d}m_{d|k-1}^{\left(j\right)},S_{d|k-1}^{\left(j\right)}\right)$
  $m_{k|k-1,d}^{\left(lJ_{k|k-1}+j\right)}=m_{k|k-1}^{\left(j\right)}+K_{k,d|k-1}^{\left(j\right)}\left(z-H_{d}m_{d|k-1}^{\left(j\right)}\right)$
  $P_{k|k-1,d}^{\left(lJ_{k|k-1}+j\right)}=P_{k|k-1}^{\left(j\right)}-P_{k,d|k-1}^{xz,(j)}{\left(S_{d|k-1}^{\left(j\right)}\right)}^{-1}{\left(P_{k,d|k-1}^{xz,(j)}\right)}^{T}$
 **end for**
 **for** $j=1,2,\ldots ,|Z_{k}|J_{k|k-1}$ **do**
  $\omega_{k|k,d}^{\left(l|Z_{k}|J_{k|k-1}+j\right)}=p_{D,d}\omega_{d|k}^{\left(j\right)}N\left(z^{\left(l+1\right)};H_{d}m_{d|k}^{\left(j\right)},S_{d|k}^{\left(j\right)}\right)$
  $m_{k|d}^{\left(l|Z_{k}|J_{k|k-1}+j\right)}=m_{k|k}^{\left(j\right)}+K_{k,d|k}^{\left(i\right)}\left(z-H_{d}m_{d|k}^{\left(i\right)}\right)$
  $P_{k|d}^{\left(l|Z_{k}|J_{k|k-1}+j\right)}=P_{k|k}^{\left(j\right)}-P_{k,d|k}^{xz,(j)}{\left(S_{d|k}^{\left(j\right)}\right)}^{-1}{\left(P_{k,d|k}^{xz,(j)}\right)}^{T}$
 **end for**

**end for**
Step 4. Weight Correction
**for** $l=0,1,\ldots ,|Z_{d}|-1$ **do**
 **for** $j=1,2,\ldots ,J_{k|k-1}$ **do**
  $\omega_{k|k-1,d}^{\left(lJ_{k|k-1}+j\right)}=\frac{\omega_{k|k-1,d}^{\left(j\right)}}{\kappa_{k}\left(z\right)+\sum_{i=1}^{|Z_{d}|J_{k|k-1}}\omega_{k|k-1,d}^{\left(i\right)}+\sum_{i=1}^{|Z_{k}\left|\right|Z_{d}|J_{k|k-1}}\omega_{k|k,d}^{\left(i\right)}}$
 **end for**
 **for** $j=1,2,\ldots ,|Z_{k}|J_{k|k-1}$ **do**
  $\omega_{k|k,d}^{\left(l|Z_{k}|J_{k|k-1}+j\right)}=\frac{\omega_{k|k,d}^{\left(j\right)}}{\kappa_{k}\left(z\right)+\sum_{i=1}^{|Z_{d}|J_{k|k-1}}\omega_{k|k-1,d}^{\left(i\right)}+\sum_{i=1}^{|Z_{k}\left|\right|Z_{d}|J_{k|k-1}}\omega_{k|k,d}^{\left(i\right)}}$
 **end for**

**end for**
Output ${\left\{\left(1-p_{D,d}\right)w_{k|k-1}^{\left(i\right)},m_{k|k-1}^{\left(i\right)},P_{k|k-1}^{\left(i\right)}\right\}}_{i=1}^{J_{k|k-1}}\cup {\left\{\left(1-p_{D,d}\right)w_{k|k}^{\left(i\right)},m_{k|k}^{\left(i\right)},P_{k|k}^{\left(i\right)}\right\}}_{i=1}^{|Z_{k}|J_{k|k-1}}\cup {\left\{\omega_{k|k-1,d}^{\left(j\right)},m_{k|k-1,d}^{\left(i\right)},P_{k|k-1,d}^{\left(i\right)}\right\}}_{i=1}^{|Z_{d}|J_{k|k-1}}\cup {\left\{w_{k|k,d}^{\left(i\right)},m_{k|k,d}^{\left(i\right)},P_{k|d}^{\left(i\right)}\right\}}_{i=1}^{|Z_{d}\left|\right|Z_{k}|J_{k|k-1}}$


## 4. Simulations

In this section, the distance which is used to measure the performance of multi-target tracking is introduced first. Then another two algorithms are given to compare with the proposed algorithm. Finally, several simulation scenarios are given to prove the effectiveness of our proposed algorithm. And simulation results show that the proposed GM-PHD using OOSM has almost the same performance as the GM-PHD using in-sequence measurement (ISM).

### 4.1. Distance

It is also an important issue to measure the tracking performance properly, which directly leads to the choice of the tracking algorithm. Improper criterion will result in tracking accuracy that cannot meet the requirement. Root mean square error (RMSE) is used to measure the distance error between estimate position and real position in a single-target tracking situation. Analogously, in a multi-target tracking scenario, RMSE is also an indicator of tracking performance. However, there is more criterion than that. For example, the accurate rate of the track associate is also an important indicator, but it does not work because the GM-PHD filter based on RFS avoids using the data association technique. Thus, Mahler [32] and Vo [33] proposed three general criteria to measure the effectiveness of multi-target tracking. The essence of these criteria for multi-target tracking performance is to measure the distance between the real state set and estimate state set.

Optimal subpattern assignment (OSPA) distance is an improvement of Wasserstein distance. The definition of OSPA distance is given as follows:

$${\bar{d}}_{p}^{\left(c\right)}\left(X,Y\right)=\left\{\begin{array}{ccc} \; & (\frac{1}{n}(\min_{\pi \in \Pi_{n}}\sum_{i=1}^{\left|X\right|}d^{\left(c\right)}{\left(x_{i},y_{\pi_{\left(i\right)}}\right)}^{p}+c^{p}\left(|Y\right|-{\left|X|))\right)}^{\frac{1}{p}}, & m\leq n\\ \; & {\bar{d}}_{p}^{\left(c\right)}\left(Y,X\right), & m>n\\ \; & 0, & m=n=0 \end{array}\right.$$

$${\bar{d}}_{\infty }^{\left(c\right)}\left(X,Y\right)=\left\{\begin{array}{ccc} \; & 0, & m\leq n\\ \; & \min_{\pi \in \Pi_{n}}\max_{1\leq i\leq n}{\bar{d}}^{\left(c\right)}\left(x_{i},y_{\pi_{\left(i\right)}}\right), & m=n\\ \; & 0, & m\neq n, \end{array}\right.$$

where $d^{\left(c\right)}=\min(c,||x-y||)$, ||·|| represents the Euler norm, *c* is the truncation distance, |X| is the cardinality of set *X*, and $\Pi_{n}$ represents all permutations of {0,1,⋯,n}. The OSPA distance can be divided into location error and cardinality error which, respectively, correspond to first item and second item of ${\bar{d}}_{p}^{\left(c\right)}\left(X,Y\right)$. Thus, it can evaluate the performance of multi-target filtering more comprehensively. It also serves the shortcomings of Wasserstein distance. And it has definition even when one of the two sets is empty or all the two sets are empty. Parameter *c* and *p* affect the weights of cardinality error and location error. How to choose the values of *c* and *p* can be refer to [33]. In general, the OSPA distance is currently recognized as an indicator of multi-target filtering.

### 4.2. Compared Algorithm

To prove the effectiveness of the proposed algorithm, two other algorithms are given here for comparison.

**Figure 4:**
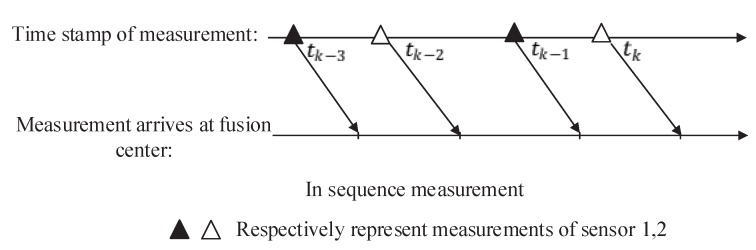
Measurements arrive at fusion center in sequence.

**Figure 5:**
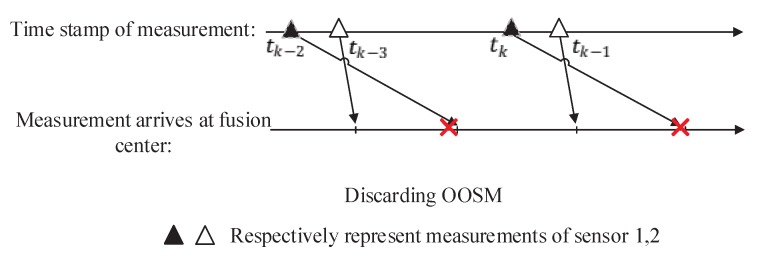
Discard OOSM when it arrives.

The first is GM-PHD using ISM. Under this circumstance, the order of arrival of two sensors at the fusion center is the same as that of sampling. Then, the sequential fusion of asynchronous measurement based on the GM-PHD filter is performed in fusion center. In another situation, the OOSM is discarded directly. Estimation of targets’ states is performed using only in sequential measurements. This situation is called Frame Drop here. The representations of these two situations are shown in Figure 4 and Figure 5.

### 4.3. Simulations Results

The simulation scenario designed here is in the 2-D plane. In the detection area of [−800, 800] m × [−800, 800] m, there are four targets appearing, all of which adopt the constant velocity (CV) model. We recall the dynamic equation as follows:

$$\begin{array}{c} x_{k+1}=F_{k}x_{k}+w_{k}, \end{array}$$

when the motion of target is CV, the transition matrix and the covariance of process noise are given as follows, respectively:

$$F_{k}=\left[\begin{array}{cccc} 1 & T & 0 & 0\\ 0 & 1 & 0 & 0\\ 0 & 0 & 1 & T\\ 0 & 0 & 0 & 1 \end{array}\right],Q_{k}=q_{v}^{2}\left[\begin{array}{cccc} T^{3}/3 & T^{2}/2 & 0 & 0\\ T^{2}/2 & T & 0 & 0\\ 0 & 0 & T^{3}/3 & T^{2}/2\\ 0 & 0 & T^{2}/2 & T \end{array}\right]$$

where $q_{v}=1\left(m/s^{2}\right)$, *T* is sampling time, and we set it to 5 s. Each target has survival probability $p_{S,k}=0.99$, and each target is detected with probability $p_{D,k}=0.98$. The observation model has been given in Equation (3). The measurement matrix and covariance of measurement noise are given as follows:

$$H=\left[\begin{array}{cccc} 1 & 0 & 0 & 0\\ 0 & 0 & 1 & 0 \end{array}\right],R_{k}=\left[\begin{array}{cc} \sigma_{x}^{2} & 0\\ 0 & \sigma_{x}^{2} \end{array}\right]$$

where $\sigma_{x}=\sigma_{y}=5$ m. Measurements are immersed in clutter, and the clutter can be modeled as a Possion RFS $K_{k}$ with intensity:

$$\begin{array}{c} \kappa_{k}\left(z\right)=\lambda_{c}Vu\left(z\right), \end{array}$$

where u(z) is the uniform density over the surveillance area, *V* is the volume of the surveillance region, and $\lambda_{c}$ is the average number of clutter returns per unit volume.

The algorithm uses the Gaussian component pruning method in the GM-PHD filter. Pruning threshold is $T_{w}=10^{-5}$, merging threshold is U=4m, and the maximum number of Gaussian component is $J_{max}=200$. Gaussian component with weights over 0.5 is considered as the estimation of state of target.

**Figure 6:**
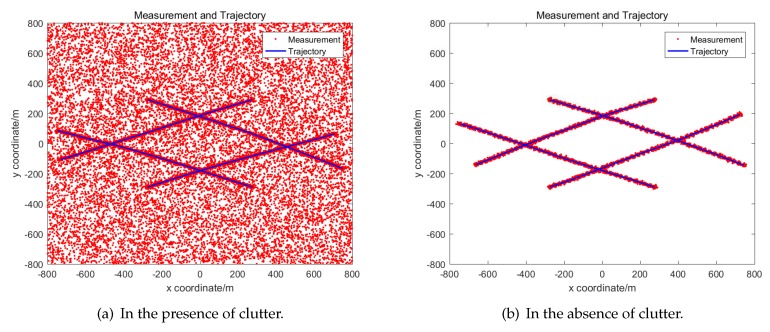
Measurements and trajectories of multi-target.

**Figure 7:**
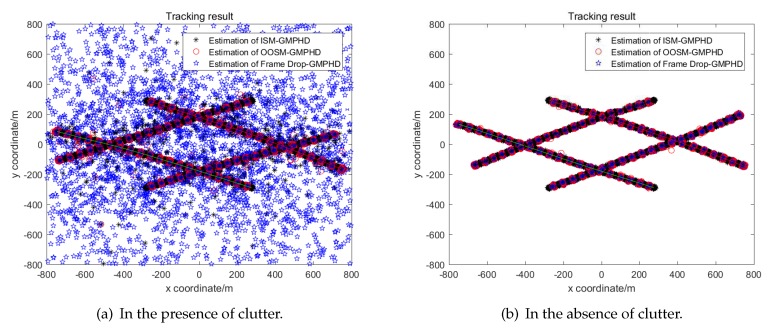
Tracking results of multi-target.

Simulation results are averaged by 100 Monte Carlo experiments. The real trajectory of each simulation is the same while measurements are regenerated each time. The real trajectory of targets and measurements of 100 simulations are shown in Figure 6. The tracking results of three algorithms are shown in Figure 7. It is obvious that the tracking performance of GM-PHD using OOSM and GM-PHD using ISM is nearly the same, while the performance of Frame Drop GM-PHD has decreased significantly.

**Figure 8:**
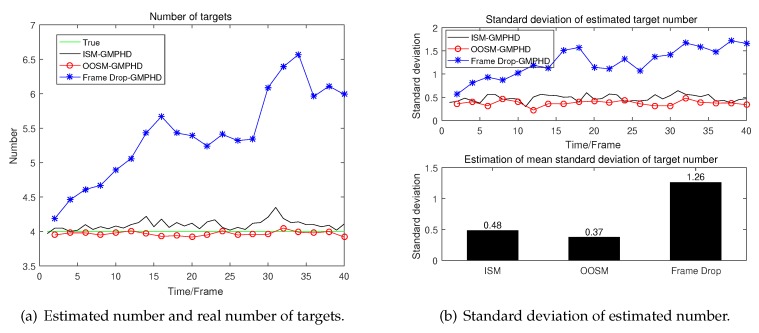
Estimated number and standard deviation in the presence of clutter.

**Figure 9:**
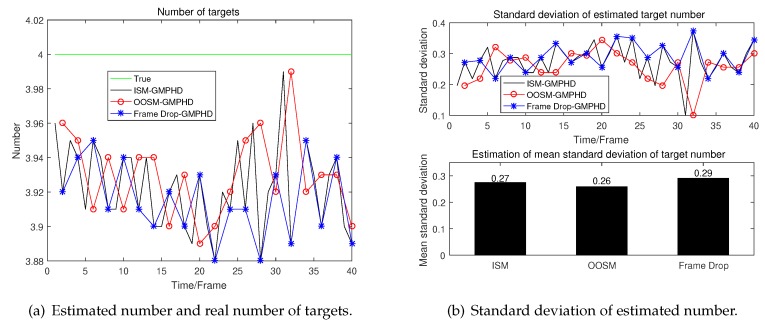
Estimated number and standard deviation in the absence of clutter.

A detailed analysis of the performance of tracking algorithms is given below. The real number of targets at different times and the number of targets estimated by different algorithms in the presence of clutter are given in Figure 8a. And the estimated number and real number in the absence of clutter is given in Figure 9a.

From Figure 8a we can see that in the presence of clutter, the result of target number estimation of GM-PHD using OOSM is even more accurate than GM-PHD using ISM. Meanwhile, the estimation of the number of GM-PHD discarding OOSM has a great deviation. And as time goes by, the algorithm even begin to diverge. The more detailed data can be seen in Figure 8b. From Figure 9, we can see that these three algorithms have almost the same performance in the estimation of number.

The simulation results of OSPA distance used to measure the results of multi-target tracking are also given as follows.

**Figure 10:**
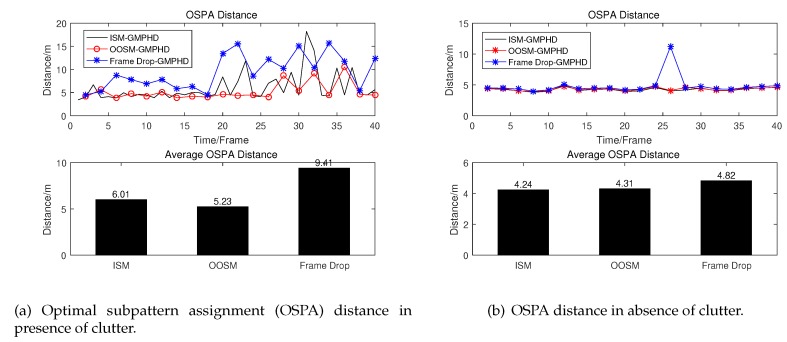
OSPA distance between real state and estimated state.

From Figure 10b, we can see that under OSPA distance criteria, our proposed algorithm which can process OOSM has almost the same performance as that of GM-PHD using ISM in the absence of clutter. However, as we all know, the clutter exists in most tracking scenario. From Figure 10a, we can see that our proposed algorithm using OOSM has better performance than GM-PHD using ISM in the presence of clutter. At the same time, in the presence of clutter situation, the performance of GM-PHD has decreased evidently when OOSMs are discarded directly. It is easy to understand that discarding OOSM directly leads to a reduction of information and consequently results in performance degradation. Compared with GM-PHD using ISM, GM-PHD using OOSM can be seen as doubling the sampling interval while the sampling information of each sample double. Due to targets are all adopting CV model, the maneuverability is not strong. Therefore, compared with the increase of information obtained by each sampling, the increase of sampling interval has little effect on the estimation accuracy. Thus, our proposed algorithm which re-updates intensity using OOSM outperforms GM-PHD using ISM in performance. Where clutter = 0 represents there is no clutter in the tracking region, and clutter = 4 represents that the average number of clutter in tracking region is 4 at each sampling time. The detailed data is shown in Table 1 which can adequately demonstrate the effectiveness of our proposed algorithm.

## 5. Conclusions

The issue of OOSM appears frequently in tracking systems. To better use the OOSM in multi-target tracking scenarios, a novel algorithm was designed in this paper. Firstly, the GM-PHD filter is introduced, and then the B1 algorithm which can solve the OOSM problem in a single-target tracking scenario is also given. Based on this, we used OOSM to re-update the posterior intensity of GM-PHD. Under the framework of the Kalman filter, we derived closed-form recursion for the intensity which is re-updated by the OOSM. Implementation of GM-PHD using OOSM was also given. Finally, several simulations were given to show that the tracking performance of our proposed algorithm is almost the same as that of GM-PHD using ISM in the absence of clutter, and the tracking performance of our proposed algorithm is even better than GM-PHD using ISM in the presence of clutter. This strongly proves the effectiveness of our algorithm. 

## Figures and Tables

**Table 1:** Tracking performance comparison of different algorithms.

	Algorithm	ISM	OOSM	Frame Drop
OSPA Distance (m)				
clutter = 0		4.24	4.31	4.82
clutter = 4		6.01	5.23	9.41
